# A lower connection to nature is related to lower mental health benefits from nature contact

**DOI:** 10.1038/s41598-024-56968-5

**Published:** 2024-03-20

**Authors:** Chia-chen Chang, Brenda B. Lin, Xiaoqi Feng, Erik Andersson, John Gardner, Thomas Astell-Burt

**Affiliations:** 1 Department of Evolution and Ecology, University of California, Davis, CA USA; 2 CSIRO Environment, GPO Box 2583, Brisbane, QLD 4001 Australia; 3 School of Population Health, Faculty of Medicine and Health, University of New South Wales, Sydney, NSW Australia; 4 Population Wellbeing and Environment Research Lab (PowerLab), Sydney, NSW Australia; 5 The George Institute for Global Health, Barangaroo, NSW Australia; 6 Ecosystems and Environment Research Programme, University of Helsinki, Helsinki, Finland; 7 Stockholm Resilience Centre, Stockholm University, Stockholm, Sweden; 8 Research Unit for Environmental Sciences and Management, North-West University, Potchefstroom, South Africa; 9 School of Architecture, Design and Planning, University of Sydney, Sydney, NSW Australia; 10 Department of Biological Sciences, National University of Singapore, Singapore, Singapore

**Keywords:** Psychology, Environmental social sciences

## Abstract

Increasing evidence demonstrates the psychological benefits of nature contact. However, the evidence is often established at the population level, and the individual differences in the psychological benefits gained from nature are considered negligible variations. In this study, we performed a cross-sectional online survey in Brisbane and Sydney, Australia, from April 15th and May 15th, 2021 around one year after the first covid-19 pandemic lockdowns. The results show that individuals with a stronger connection to nature are linked with a lower level of stress and anxiety with increased frequency in public greenspace visits, while such an association is less clear for individuals with a weaker connection to nature. We also find that, through the answer to an open-ended question, individuals with a lower connection to nature tend to mention nature-related words less as the reason for visiting greenspace. This indicates that a person’s connection to nature is linked with how they interact with nature and thus might determine whether and how much psychological benefit a person gains from experiencing nature.

## Introduction

A large body of evidence shows contact with nature (e.g., parks, forests) can be beneficial for mental health and wellbeing^1–3^. A person’s connection to nature, positive feelings and attitudes towards nature are positively correlated with nature contact^4–6^. Connectivity with nature describes a connection between the self, others, and the natural world^7^. Given a similar amount of nature contact, people with a stronger connection to nature may also gain more psychological wellbeing benefits from these nature contact^8^. People of different cultural upbringings manifest different inclinations towards nature^9–11^. For example, the Menominee People of Wisconsin, USA were more likely to perceive themselves as part of nature compared to their European American counterparts^10^. Individual differences in the connection to nature have also been found to be associated with both genetic and environmental components based on a twins analysis^12^. The differences in how a person perceives and interacts with nature potentially affect the extent which individuals benefit from the nature contact.

It remains largely unexplored whether and how individual differences in one’s connection to nature may shape the relationship between nature contact and psychological benefits. Mechanisms in the relationship between nature contact and psychological wellbeing include attention restoration and stress reduction^13,14^. The theories behind both postulate that nature has restorative and stress-reducing benefits for people^3^. Stress reduction theory suggests that innate affinity to nature contributes to positive emotions after experiencing nature^14^, suggesting a person’s affinity to nature is the key to generate wellbeing benefits. More specifically, the nature preference hypothesis^15^ suggests that mental health from nature is realised because nature is often the preferred environments over urban environments for individuals. If this is true, individuals with a stronger connection to nature may receive greater improvements in their psychological wellbeing when exposed to nature^16–18^. For example, a survey of greenspace visits and life satisfaction from Singapore showed that people with a higher connection to nature that spent more than one hour in natural spaces per week reported higher life satisfaction, but this relationship was less clear for people with a low connection to nature^8^. In another example, people with higher connection to nature benefit from mood improvements from viewing nature images^15^ or lab-based immersive simulation^19^. However, it has also been shown that individuals with higher nature connection tended to have higher Eudaimonic wellbeing, but the increased in frequency of nature contact did not improve the wellbeing as much as for individuals with low nature connection who tended to have lower wellbeing^20^. The other line of theory suggests that nature can improve our cognitive ability because natural environments are less cognitively demanding than urban environments (i.e., attention restoration hypothesis), a recent study found that individuals with more nature contact had greater attentiveness regardless of that individual’s connection to nature^21^. It is important to note that attention restoration hypothesis focuses on cognitive wellbeing. Only a handful of studies investigated the individual differences in the impact of natural environments on wellbeing; therefore, it is unclear if the connection to nature influences the relationship between nature contact and wellbeing benefits.

In this study, we focus on how one’s connection to nature may translate into wellbeing outcomes through greater attentiveness to nature around specific individuals. We hypothesize that the relationship between wellbeing and nature contact is stronger for people with a stronger connection to nature than people with a weaker connection to nature. This could be because individuals with a stronger connection to nature tend to be more conscious and more mindful of the nature around them when they visit such spaces. Mindfulness, among other factors, may then influence how a person perceives nature. For example, it has shown that perceived biodiversity, which widely varies among individuals, explains the wellbeing benefits gained better than the actual biodiversity in a location^22,23^. Thus, the relationship between nature contacts and wellbeing benefits gained could also be highly subjective and shaped by a person’s connection to nature through their interaction with nature. It is likely that individuals with a stronger connection to nature tend to use the natural environments more intentionally and actively observe and engage with natural elements when in public green spaces.

We performed an online greenspace use and lifestyle survey in Brisbane (n = 1050) and Sydney (n = 1034), Australia. Sydney is the capital of New South Wales and the most densely populated city in Australia. The Greater Sydney area has about 22% tree cover, with the inner city area of Sydney providing 27 m square of green space per person^24,25^. Brisbane is the capital of Queensland and the third most populous city in Australia. The metropolitan area exhibits high overall levels of public greenspace and tree cover (36%), with the inner urban area providing about 48 m square of green space per person^26^. In these two cities, we have shown that more frequent private yard visits was associated with better wellbeing^27^, and individuals who spent more time in public greenspaces than a year prior or with stronger nature orientation experienced obvious improvements in their health and wellbeing^28^. In this study, a cross-sectional correlation was done, and the data were collected from April 15th and May 15th 2021 through an online data collection company with each participant answering the survey once. The survey questions include the duration and frequency of public greenspace and private yard visits, connection to nature, which was measured using a subscale of Nature Relatedness (i.e., NR-Exp) to specifically quantify a person’s familiarity to nature and desire to be in nature. The level of stress, anxiety, and depression was measured using DASS-21 scale. The respondents were also asked their reasons to visit public and private greenspaces.

We aim

1. To examine how individual’s connection to nature interacts with the frequency and duration of public greenspace visits and those of private yard visits in predicting an individual’s mental health, specifically stress, anxiety, and depression.
2. To understand how individual’s connection to nature is associated with their reasons to visit green spaces and attentiveness of nature when visiting public greenspace.

## Results

### Visiting greenspace links with lower stress, anxiety, and depression, positively moderated by connection to nature

**Figure 1:**
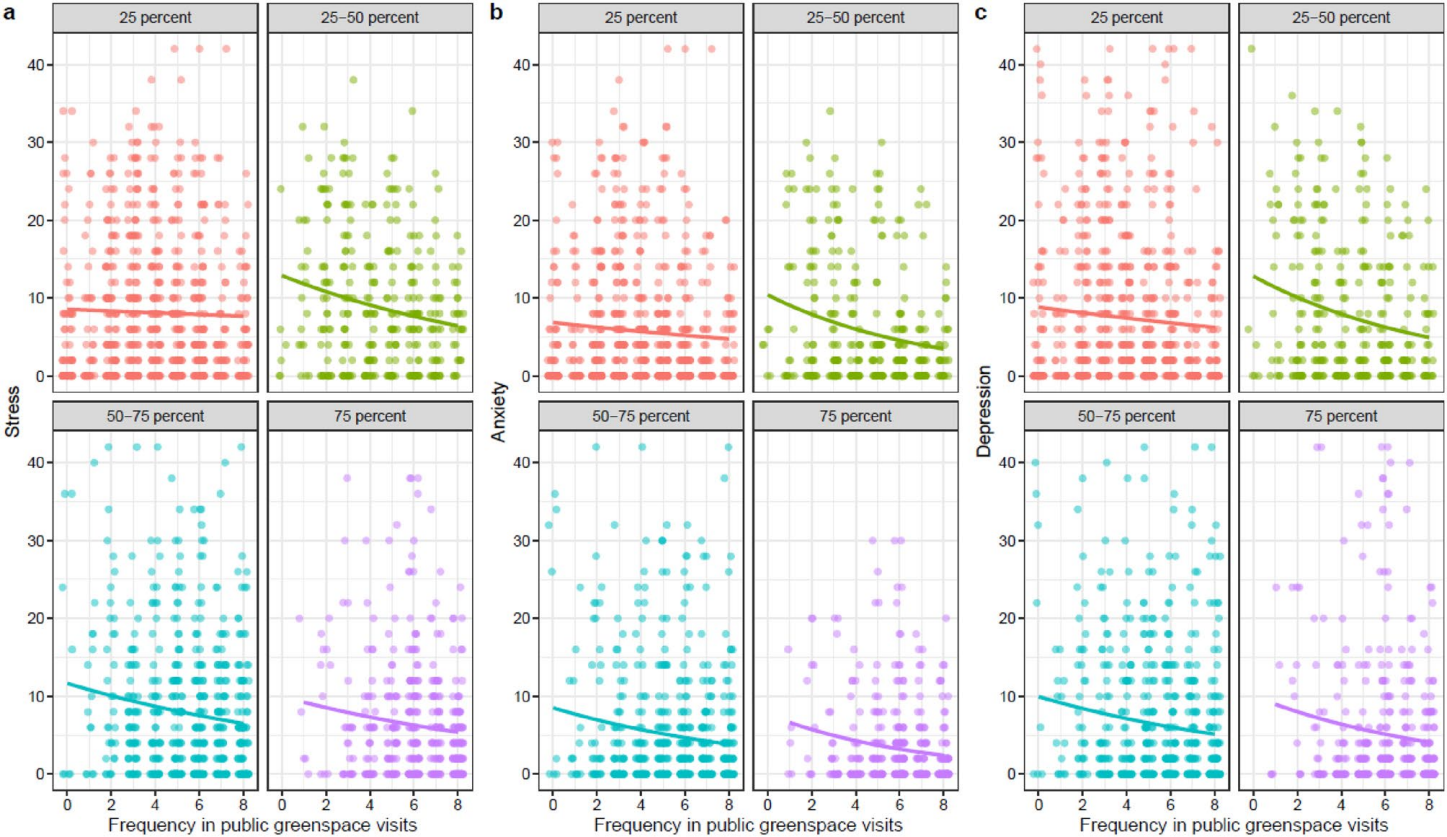
Patterns of stress, anxiety, and depression in relation to the frequency of public greenspace visits. The graphs are separated into quartiles of connection to nature: bottom 25 percentile (red, equal or less than 3 connection to nature score), 25 to 50 percent (green, from 3 to 3.33), 50 to 75 percent (blue, from 3.33 to 4), and top 25 percentile (purple, more than 4) of participants. Quartiles were used for visualization purposes. In the analysis, actual scores of a person’s connection to nature were used in the statistical analyses.

We find that on average, people who visit public greenspace more often had lower stress, anxiety and depression scores than people who visit public greenspace less often (Table 1). However, when we consider the variation of an individual’s connection to nature, the reduction in stress, anxiety, and depression varied depending on a person’s connection to nature (Table S1, Table S2, Table 1). Specifically, people with a weak connection to nature (the lowest quartile of the connection to nature score) exhibited little reduction in stress and anxiety scores even when they visited public greenspace frequently (Fig. 1). Analysis of depression scores showed a similar trend as the stress and anxiety measures, with a lower effect for individuals with a low connection to nature, though the interaction effect was not statistically significant. More information on the comparison of model with different interaction terms can be found in the online supplementary note 1.

**Table 1:** The summary of the best generalized linear model for predicting stress, anxiety, and depression, which includes the interaction between the connection to nature and the frequency of greenspace visits (Connection to nature x Frequency of greenspace visits).

	Stress			Anxiety			Depression		
	Estimate	Standard error	*p*-value	Estimate	Standard Error	*p*-value	Estimate	Standard error	*p*-value
(Intercept)	2.879	0.108	< 0.001	2.748	0.138	< 0.001	2.960	0.132	< 0.001
**Frequency of yard visits**	**− 0.111**	**0.038**	**0.004**	**− 0.104**	**0.050**	**0.040**	**− 0.163**	**0.047**	**0.001**
**Duration of yard visits**	**0.156**	**0.038**	**< 0.001**	**0.233**	**0.049**	**< 0.001**	**0.185**	**0.047**	**< 0.001**
**Frequency of greenspace visits**	**− 0.086**	**0.028**	**0.002**	**− 0.168**	**0.037**	**< 0.001**	**− 0.110**	**0.035**	**0.001**
Duration of greenspace visits	**− **0.013	0.027	0.618	**− **0.012	0.035	0.738	**− **0.056	0.034	0.101
Connection to nature	0.001	0.028	0.963	**− **0.065	0.037	0.078	**− **0.024	0.035	0.485
**Age**	**− 0.105**	**0.008**	**< 0.001**	**− 0.149**	**0.010**	**< 0.001**	**− 0.102**	**0.009**	**< 0.001**
Gender (male)	**− **0.068	0.049	0.168	0.001	0.064	0.984	**− **0.018	0.061	0.773
Income	**− **0.017	0.009	0.070	**− 0.035**	**0.012**	**0.003**	**− 0.055**	**0.011**	** < 0.001**
**City (Sydney)**	**0.167**	**0.049**	**0.001**	**0.375**	**0.065**	**< 0.001**	**0.214**	**0.061**	**< 0.001**
Education	**− **0.006	0.010	0.535	**− **0.017	0.013	0.188	**− **0.013	0.012	0.300
**Connection to nature x Frequency of greenspace visits**	**− 0.081**	**0.026**	**0.002**	**− 0.111**	**0.035**	**0.002**	**− **0.052	0.032	0.102

Note that the nature contact measurements and connection to nature were mean-centred. Statistically significant variables are highlighted in bold.

### Individuals with stronger connection to nature are more conscious of nature

To gain a greater understanding of why individuals with differing level of connection to nature have a different response in the reduction in the stress, anxiety, and depression from the frequency of greenspace visits, we performed text-analysis on an open-ended question specifically asking respondents to list multiple reasons why they visit public greenspace. We calculated proportion of answers being related to nature or other activities for each individual. Individuals who answered this question with mostly nature-related words may use nature as a primary focus for their visit, thus being intentional about going to the greenspace for nature itself, whereas individuals who list mostly other activities as the reasons may use nature as background for other primary activities drawing them to greenspaces (e.g. for physical activity or socialising) and less intentional about nature itself.

**Figure 2:**
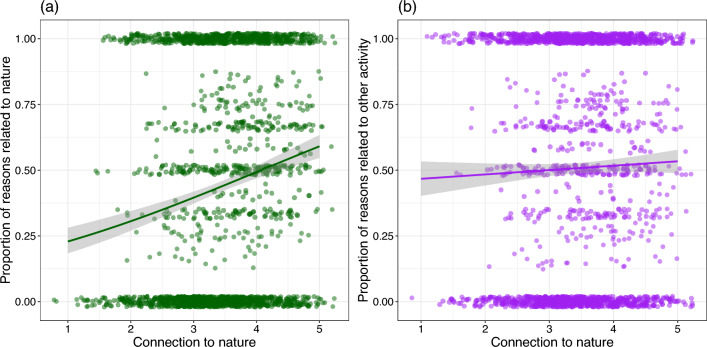
Results from text analysis for reasons to visit greenspace (i.e., more or less conscious of nature). Survey respondents were asked to list at least one reasons they visit public greenspaces, and the answers were then categorized as related to nature (i.e., more conscious of nature, e.g., tress, birds, panel **a**) or to other activities (i.e., less conscious of nature, e.g., picnic, exercise, panel **b**). Each answer could be either nature or other activities, or both (e.g., walk and enjoy wildlife) or none of the category (e.g., big and good). The proportion of answers in each category were then calculated for each respondent (1 being 100% of the answer that given by a participant is classified as that particular category). In each panel, each point is one individual. Individuals with a stronger connection to nature more often mention nature-related words when asked their reasons to visit a public greenspace (panel a), while there is no such pattern for reasons related to other activities (panel b).

We found that individuals with a stronger connection to nature tend to mention nature-related words in response to this survey question as compared to individuals with a weaker connection to nature (e.g. water, bird, forest; Fig. 2, Table 2), suggesting they are likely to be more intentional about nature when they visit public greenspaces and attentive to the qualities that sets nature apart from other places for social-recreational activities. However, individuals with either high or lower connection to nature are equally likely to mention words such as picnic, bike, and market, indicating that nature may play a role in the background of other activities (Fig. 2, Table 2). Regardless of the connection to nature, all individuals who are using these spaces are being exposed to nature, but those with a stronger connection to nature are visiting with a greater intention to nature qualities of greenspaces. The text-analysis focusing on private greenspace visits is shown in Table S3 and shows similar results to that of the public greenspace analysis.

**Table 2:** Generalized linear models with quasibinomial error structure show the association between a person’s connection to nature and proportion of answers with nature-related words, but not associated with proportion of answers with other activities.

Words related to nature directly				Words related to other activities or reasons			
Bag of stem words: view, tree, water, green, natur, river, beach, plant, bird, bush, flower, sceneri, grass, wildlif, greeneri, lake, anim, bushland, sea, ocean, "flora", "pond", duck, scenic, creek, waterfal, forest, rainforest, fish, fauna, bay, landscap, mountain, rose, veget, hill, land, garden, park, bushwalk				Bag of stem words: walk, close, home, dog, track, play, kid, playground, path, picnic, friend, bike, facil, exercis, run, cafe, peopl, activ, conveni, access, equip, proxim, famili, children, sport, bbq, cycl, swim, walkway, sit, ride, seat, court, hike, leash, basketbal, coffee, shop, toilet, amen, golf, pool, use, food, divers, market, children, work, traffic, crowd, game, near, swing			

	Estimate	Standard Error	*P*-value		Estimate	Standard Error	*P*-value
**(Intercept)**	**− 1.660**	**0.246**	**< 0.001**	(Intercept)	**− **0.010	0.244	0.966
**Connection to nature**	**0.383**	**0.054**	**< 0.001**	Connection to nature	**− **0.031	0.054	0.567
**Age**	**0.061**	**0.012**	**< 0.001**	**Age**	**0.030**	**0.012**	**0.011**
**Gender (male)**	**− 0.331**	**0.079**	**< 0.001**	**Gender (male)**	**− 0.318**	**0.080**	**< 0.001**
Income	**− **0.027	0.015	0.075	Income	0.015	0.015	0.337
Education	0.022	0.016	0.175	Education	0.016	0.016	0.337
**City (Sydney)**	**− 0.180**	**0.078**	**0.021**	City (Sydney)	**− **0.142	0.078	0.071

The bag of words is listed as stem words generated using Porter’s stemming algorithm (e.g., both nature and natural will be converted to natur). Noted that not all the words are able to be classified into one of the categories, such as big or area. Statistically significant variables are highlighted in bold.

## Discussion

A person’s connection to nature plays a moderating role in an individual’s mental health response to the frequency of spending time in nature. Specifically, visiting public greenspace frequently reduced the levels of stress, anxiety, and depression for people in general, while with the average level of frequency of public greenspace visits, the connection to nature alone is not associated with the levels of stress, anxiety, and depression. More importantly, these positive benefits from nature contact may be less clear in people who have a low connection to nature, which in this study represents the bottom 25 percentile of our sampled population. On the other hand, people with a stronger connection to nature gain more benefits from nature contact as the relationship between these individuals’ connection to nature and mental health score were more pronounced. We further show that a potential underlying factor for this different effect may be that individuals with a higher connection to nature may also be more attentive to nature itself as they often listed nature as their reasons to visit nature.

Although many studies have shown that spending time in greenspace can have a positive relationship with mental health^1,2,27,29^, few studies have attempted to study the moderating effect of nature orientation on mental health benefits from greenspace visitation. We showed that individuals with a low connection to nature may experience the wellbeing effects not to a similar degree even though they visit public greenspaces frequently. The variability of benefit individuals may receive was highly related to differences in personal outlooks, in this case the connection to nature, with the lack of improvement in the subjective wellbeing in individuals with a low connection to nature per se. This result may be because these individuals, even when they spend a lot of time in nature, are not conscious of nature, and therefore do not receive the wellbeing benefit from nature experience than people who are more conscious of nature. We hypothesise that this lack of improvement may be because of a less intentional interaction with or active attention to nature by these individuals even for those visit greenspace frequently. We found that individuals with a strong connection to nature were likely to be more intentional with nature itself when they visit public greenspace than people with a low connection to nature. This suggests that to maximize the services from the availability of public greenspace for urban residents, other factors, such as connection to nature and the engagement of nature environment, likely will lead to better mental health benefits.

In our study, we treat a person’s connection to nature as relatively stable trait. However, a person’s connection to nature may change because of their experiences, such as greenspace around their houses or influences from their friends and families^30,31^. Factors that improve one’s connection to nature should be considered and investigated. It is important to note that a person’s connection to nature could change at different life stages. For example, education sectors can provide engaging nature education for children, which has been shown to be associated with connection to nature as adults. It has also been shown that individuals who live near to greenspace tend to be more connectedness to nature^31^. The urban development sector could increase high-quality coverage of greenspaces in residential areas with social programs that help teach people how to engage with nature when in public greenspaces^32–34^. These investments may provide the supports to individuals to learn new ways to interact with nature or be mindful of natural elements in public green spaces even when the initial main goal of going to a public greenspace is a non-nature based activity^35^. Therefore, urban planners could consider how to design public greenspace that enhances the direct interaction with nature and creates opportunities for purposeful nature interaction^36,37^. As our study is a correlative study, future studies could perform experiments that manipulate the level of engagement with nature while controlling for one’s connection to nature to test whether improving the nature engagement alone would yield mental wellbeing benefits.

Other personal factors may also be highly relevant in the potential to gain wellbeing benefits. People who visited their private yard more often also received greater mental health benefits, in line with previous evidence of the benefits of spending time in private yard space for mental health^38,39^. This finding may be associated with the other socio-demographic factors from the analysis showing older and wealthier individuals having better mental health. Older and high-income individuals may have more time to spend in high-quality greenspaces, and they have fewer economic stresses to contend with due to their economic privilege^40^. The finding that individuals in Sydney tend to experience higher levels of stress and anxiety may be related to a number of factors in addition to differences in the land use, such as income and cost of living in Sydney being approximately 10% greater than that of Brisbane; however, it is unclear that the difference in the greenspace coverage between two cities can explain the mental health status difference; in-depth research is required to understand the reasons why this pattern has been seen.

It is important to note that this is a cross-sectional study; we were unable to draw causality from this study as it provided primarily a correlative understanding of relationships. One possibility is that individuals who visit greenspace more often tend to have better welling. An alternative explanation will be that people with better mental health status may visit greenspaces more often. It is difficult to draw this conclusion with the data presented. In addition, our study did not find evidence of relationship between duration of public greenspace visits and the level of a person’s mental health. It is important to note the duration of public greenspace visits was considered only within the week prior to the survey and represents a short and recent period of use. This may be confounded by the observation that some individuals decided to spend more time in public greenspace for emotional regulation because they were suffering from stress, anxiety, or depression around the time of the survey collection^27^. Our study did not allow us to tease apart from these possible causal relationships. Future studies could consider a longitudinal study or empirical studies to draw a clearer understanding of the causality of the patterns observed.

In addition, our study did not consider the activities that people actually do in the greenspaces. When people spend time in a public greenspace, they may intentionally engage with nature (e.g., bird watching) or use green space as a backdrop for another activity (e.g., exercise). The different ways of using green spaces could be an underlying factor in shaping the relationship between nature experience and wellbeing. Future studies could examine whether people with different levels of connection to nature use greenspace in different ways, such as intentionality or level of engagement with nature, which may indirectly contribute to the wellbeing outcomes. Our study focuses on a person’s physical familiarity with nature and desire to be in nature for a person’s connection to nature. However, a person’s connection with nature is complex and includes multiple aspects, such as a person’s self-identity with nature, concern of human’s impact on biodiversity, and environmental values^7,41–44^. A person’s environmental value may be negatively associated with wellbeing due to eco-anxiety or climate anxiety^45,46^. Future study could investigate the moderating effect of other aspects of nature relatedness on the relationship between nature contact and wellbeing. Lastly, our data collection was carrying out during COVID-19 pandemic period, which may introduce some nuances although the similar results have been reported before COVID-19.

In conclusion, our results highlight the variation across individuals in terms of the wellbeing benefits that a person can gain from nature contact. Although, this area of study has become a significantly researched field in the last decade, there are still many complexities to understanding how an individual’s connection to nature can change over time and through situational circumstances. In this paper, we have found that there may be a link between one’s connectedness to nature, their intentional use of green space, and the well-being benefits received. This data allows for this level of complexity to be understood, however, change through time and context is still an important longitudinal process to understand—especially as interventions can affect changes in one’s nature relatedness through time. Considering that mental health care needs are increasing globally, a better understanding of the role of greenspace and how it interacts with the diversity of traits within a community to provide benefits will be important for the future design of communities and neighbourhoods.

## Methods

We performed an online survey in Brisbane and Sydney, Australia from April 15th and May 15th, 2021 through an online data collection company, the Online Research Unit. A total of 1050 respondents were collected from Brisbane and 1034 surveys were collected from Sydney for a total of 2084 responses. To obtain a demographically representative sample, demographic parameters were provided to the online data collection company to ensure that sampling occurred across gender, education, and income variable to be representative of each city. A minimum high quality sampling number was requested from the survey company based on the demographic parameters. Discussions with company representative statisticians with the project team were conducted to determine that a sample of n = 1000 would provide enough respondents in each category across demographic variables for sufficient power in statistical analyses.

In these two cities, we examine how an individual’s connection to nature interacts with the frequency and duration of public greenspace visits and those of private yard visits in predicting an individual’s mental health, specifically stress, anxiety, and depression. To understand their intentionality in the use of greenspace, the respondents were also asked their reasons for visiting public greenspaces, which were then classified as either nature relate and/or other related to other activities. This research was conducted in accordance with approved guidelines, and all protocols were received under Institutional Human Research Ethics Approval (CSIRO Human Research Ethics Review Board, Project 144/20). Informed consent was obtained from all respondents.

To account for potential socio-demographic factors, socio-demographic information was also collected which were age, gender, education, and income. The options in each demographic information and the sample size are shown in the supplementary note 2. The full item to measure connection to nature is also included in the supplementary note 2. The full questions are included in supplementary note 3.

### Assessment of greenspace visits

Nature contact include four measurements: frequency of public greenspace visits, frequency of private yard visits, duration of public greenspace visits last week, and duration of private yard visits last week. It is important to note that the two measurements—frequency and duration—represent different aspects of greenspace visitation in terms of timing and intensity of use. The frequency measurement asks respondents how often in general they use greenspace over the course of a year. The duration measure specifically focuses on the amount of time spent over the course of the week before they respond to the survey.

For public greenspace visits, participants were asked to recall about how often they usually visit or pass through outdoor greenspaces for any reason. The frequency was selected from the following categories: never (= 0), once a year (= 1), once every three months (= 2), once a month (= 3), 2–3 times a month (= 4), once a week (= 5), 2–3 days a week (= 6), 3–5 days a week (= 7), and 6–7 days a week (= 8). Participants were also asked to recall over the last week what outdoor greenspaces they visited or travel through and to estimate the total time (hours) they spent there. Participants who reported spending more than 168 h or less than 0 h in public greenspaces last week were considered an error, and 9 participants were removed from the analysis for this reason (n = 2075). Participants who spent more than 10 h in public greenspaces last week were coded as 10 h. It is important to note the duration and frequency of public greenspace visits were measured at different time intervals (i.e., over a year vs last week).

For private yard visits, similarly, participants were asked to recall how often they usually spend more than 10 min in their own yard or on their deck. The frequency was selected from the following categories: never (= 0), less than once a month (= 1), 2–3 times a month (= 2), once a week (= 3), 2–3 days a week (= 4), 4–5 days a week (= 5), and 6–7 days a week (= 6). Participants who chose “I don’t have a yard or deck” (n = 248) were considered as having zero frequency in yard visits. This is because people who do not have a yard or deck did not have any time spent in their own yard. Participants were also asked to think about the last week, and how much time in total they spent in their own yard or on their deck. The duration was selected from the following categories: No time (= 0), 1–30 min (= 1), 31 min to 1 h (= 2), 1–3 h (= 3), 3–5 h (= 4), 5–7 h (= 5), 7–9 h (= 6), more than 9 h (= 7). Similar to the frequency of yard visits, participants who chose “I don’t have a yard or deck” were considered as having a zero duration of yard visits last week.

### Assessment of a person’s connection to nature and psychological wellbeing

We used a subscale in the Nature Relatedness Scale to assess participants’ level of connection to nature^41^. The Nature Relatedness Scale consists of 21 statements, which includes three subscales, which are identification with nature (NR-self), a sense of agency related to human’s impact on all living things (NR-perspective), and a physical familiarity and a desire to be out in nature (NR-experience). Because we aimed to test the influence of a person’s desire to be in contact with nature, we isolated NR-experience consisting of 6 statements on a scale from strongly disagree (= 1) to strongly agree (= 5). A person with a higher average score across the 6 statements of NR-experience has a stronger familiarity and desire to be out in nature. The three sub-scales are allowed to use independently^41^, and the NR-experience has been shown to have good internal consistency (Chronbach’s alpha = 0.8; current sample with Chronbach’s alpha = 0.73 using ltm package^47^) and temporal stability within an individual (correlation coefficient = 0.85 over a 6-to-8-week period)^41^.

We used the Depression, Anxiety and Stress scale (DASS-21) consisting of 21 items, with 7 items per subscale (for depression, anxiety, and stress)^48^ to assess one’s psychological wellbeing. Participants were asked to score every item on a scale from *did not apply to me at all* (= 0) to *applied to me very much* (= 3). We summed scores per subscale and multiplied them by a factor of 2 based on the scale development processes described in^48^. A person with a higher score is more stressed, anxious, and depressed.

### Text classification analysis: reasons for using public and private greenspaces

To understand whether individuals with different levels of connection to nature have different reasons to use public greenspaces, the respondents were asked to list the name of parks that they visit most often and what are the aspects or factors that drive them to use these parks. They were open-ended questions with up to 7 reasons. We used tm and SnowballC libraries in R v4.2.2 to perform text classification^49–51^. We first use tm_map to remove numbers, stop words (e.g., ‘I’ or “the”), and punctuations, and organize stem words (e.g., both nature and natural will be converted to natur). We then selected the most commonly used 200 words to create “bag of words”. The classification of the words was done by one author (CC). The nature-related words include such as tree, nature, wildlife, and activity related include, such as walk, play, kid, swim. We then classified whether each reason provided by each respondent contained “nature” or “activity” words. As the answers were diverse, which could be both one world or several words in one field, each answer it could also be categorized in both (e.g., walk and enjoy wildlife) or none of the category (e.g., big and good). The proportion of reasons for nature and activity were then calculated for each respondent.

For reasons to use private greenspaces, the respondents were asked in an open-ended question to describe what aspect they like about their private greenspace (i.e., yard) and what makes they want to spend time in it? The analysis procedure is similar to the one to analyse reasons to use public greenspace, as above.

### Statistical analyses

All analyses were conducted in R v4.2.2^52^. We ran three sets of analyses for each mental health condition: stress, anxiety, and depression. In each set of analysis, we used generalized linear models with a quasi-Poisson error structure to account for overdispersion. The explanatory variables were frequency of yard visits, duration of yard visits last week, frequency of public greenspace visits, duration of public greenspace visits, connection to nature, age, gender, city, income, and education. Nature contact measurements and connection to nature were mean-centred.

To examine whether people with a stronger connection to nature are more sensitive to the nature contact, in each set of analysis, we ran five different models with four models with an interaction term between connection to nature and one measurement of nature contact (connection to nature x frequency in greenspace visits, connection to nature x duration in greenspace visits, connection to nature x frequency in yard visits, connection to nature x duration in yard visits) and one model not having any interaction term. We then performed model comparisons. In the model comparison, we ran models with Poisson error structure and qAIC (quasi-Akaike Information Criterion) of each model was calculated considering overdispersion parameters, and qAIC was then used to compare models using package *bbmle*^53,54^. The parameter estimates from best models were obtained using a quasi-Poisson error structure. The multi-collinearity of variables was checked using vif function in package *car* with vif 3 as a cut-off^55^.

To examine whether a person’s connection to nature associates with their reasons to visit public green spaces, we ran two generalized linear models with quasibinomial to account for overdispersion (one for the proportion of nature-related reasons, and the other for the proportion of other activity reasons), and the predictors are connection to nature, age, gender, income, education, and city. We also ran similar analyses to understand whether a person’s connection to nature links with their reasons to use yards. We ran one generalized linear model for reasons to use private greenspace being related to nature directly, and the other one for reasons being related to other activities with binary response variables and predictors were identical as those to analyse public greenspace.

### Supplementary Information

Supplementary Information.

## Data Availability

The datasets aggregated and/or analysed during the current study are available from the corresponding author on reasonable request.
