# Supplementary material for: A lower connection to nature is related to lower mental health benefits from nature contact
Source: Sci Rep. 2024 Mar 20;14:6705. doi: 10.1038/s41598-024-56968-5 (PMC10954714; doi:10.1038/s41598-024-56968-5)
Supplement: Supplementary file 1 — Supplementary Information. [file 41598_2024_56968_MOESM1_ESM.docx]

**Supplementary materials**

**Supplementary note 1.**

Our second-best model is the one that includes the interaction term between the duration of greenspace visits last week and the connection to nature (Table S1). The duration of greenspace visits did not have a significant association with mental health for people with an average connection to nature; however, it is linked with lower stress and anxiety for people with a stronger connection to nature but linked with higher stress and anxiety for people with a weak connection to nature (Table S2, Figure S1).


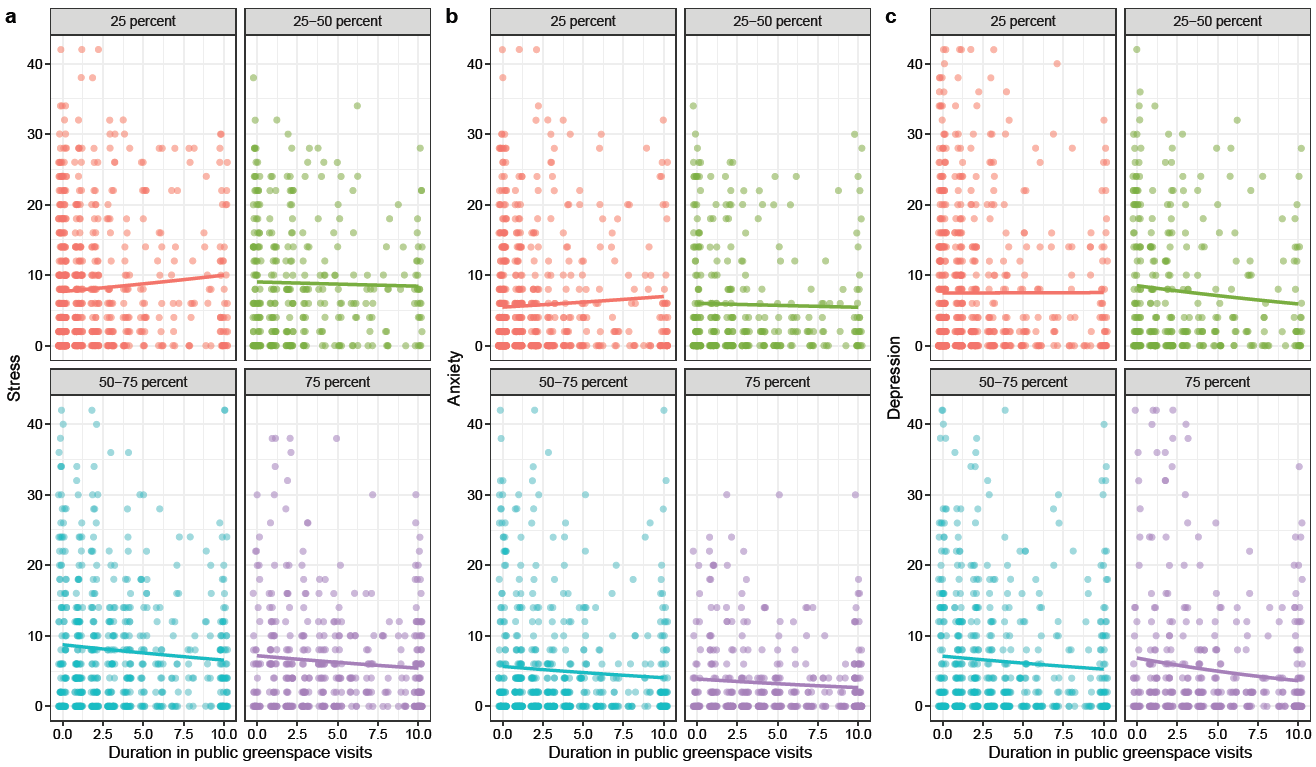


**Figure S1.** Patterns of stress, anxiety, and depression in relation to the duration of public green space visits. The graphs are separated into quartiles of connection to nature: bottom 25 percentile (red), 25 to 50 percent (green), 50 to 75 percent (blue), and top 25 percentile (purple) of participants. Noted that the quartiles were used for visualization only, and the actual scores of a person’s connection to nature were used in the statistical analyses.

**Table S1.** The comparison of models in predicting stress, anxiety, and depression. The only difference between models are the interaction terms. All models contained all four measurements of nature experience, connection to nature, age, sex, income, education, and city.

|  | Stress | | Anxiety | | Depression | | |
| --- | --- | --- | --- | --- | --- | --- | --- |
|  | dqAIC | weight | dqAIC | weight | dqAIC | weight |  |
| Connection to nature x frequency in greenspace visits | 0.00 | 0.5322 | 0.0 | 0.887 | 0.0 | 0.295 |  |
| Connection to nature x duration in greenspace visits | 0.4 | 0.4436 | 5.1 | 0.071 | 0.4 | 0.239 |  |
| Without interaction term | 7.6 | 0.0120 | 8.1 | 0.015 | 0.7 | 0.207 |  |
| Connection to nature x duration in yard visits | 8.9 | 0.0063 | 8.3 | 0.014 | 1.1 | 0.170 |  |
| Connection to nature x frequency in yard visits | 9.0 | 0.0059 | 8.3 | 0.014 | 2.4 | 0.088 |  |

**Table S2.** The summary of the second-best model. Note that the nature experience measurements and connection to nature were mean-centred.

|  | Stress | | |  | Anxiety | | |  | Depression | | |
| --- | --- | --- | --- | --- | --- | --- | --- | --- | --- | --- | --- |
|  | Est | SE | p-value |  | Est | SE | p-value |  | Est | SE | p-value |
| (Intercept) | 2.863 | 0.109 | <0.001 |  | 2.729 | 0.139 | <0.001 |  | 2.950 | 0.132 | <0.001 |
| Frequency of yard visits | **-0.110** | **0.038** | **0.004** |  | **-0.102** | **0.051** | **0.045** |  | **-0.162** | **0.048** | **0.001** |
| Duration of yard visits | **0.152** | **0.038** | **<0.001** |  | **0.230** | **0.050** | **<0.001** |  | **0.183** | **0.047** | **<0.001** |
| Frequency of greenspace visits | **-0.080** | **0.028** | **0.004** |  | **-0.151** | **0.037** | **<0.001** |  | **-0.104** | **0.034** | **0.003** |
| Duration of greenspace visits | -0.006 | 0.027 | 0.833 |  | -0.012 | 0.035 | 0.738 |  | -0.053 | 0.034 | 0.123 |
| Connection to nature | 0.006 | 0.028 | 0.823 |  | -0.052 | 0.037 | 0.160 |  | -0.021 | 0.035 | 0.542 |
| Age | **-0.105** | **0.008** | **<0.001** |  | **-0.150** | **0.010** | **<0.001** |  | **-0.102** | **0.009** | **<0.001** |
| Gender (male) | -0.069 | 0.049 | 0.160 |  | -0.001 | 0.065 | 0.987 |  | -0.018 | 0.061 | 0.766 |
| Income | -0.016 | 0.009 | 0.083 |  | **-0.034** | **0.012** | **0.005** |  | **-0.055** | **0.011** | **<0.001** |
| City (Sydney) | **0.171** | **0.049** | **<0.001** |  | **0.380** | **0.065** | **<0.001** |  | **0.217** | **0.061** | **<0.001** |
| Education | -0.006 | 0.010 | 0.565 |  | -0.017 | 0.013 | 0.202 |  | -0.013 | 0.012 | 0.314 |
| Connection to nature x Duration of greenspace visits | **-0.077** | **0.025** | **0.002** |  | **-0.076** | **0.034** | **0.025** |  | -0.049 | 0.032 | 0.131 |

**Table S3.** Generalized linear models show the association between a person’s connection to nature and nature-related reasons to visit private greenspaces, but not associated with other activities. The bag of words is listed as stem words (e.g., both nature and natural will be converted to natur). Noted that not all the words are able to be classified into one of the categories, such as big or area.

| Words related to nature directly | | | | Words related to other activities or reasons | | | |
| --- | --- | --- | --- | --- | --- | --- | --- |
| Bag of stem words:  plant, tree, grass, green, bird, view, lawn, flower, natur, veget, greeneri, fruit, bush, shrub, weed, vege, wildlif, bee, herb, grassi, landscap, leafi, insect, anim, rose, tropic,  bildlif, cat, creek | | | | Bag of stem words:  pool, sit, quiet, dog, play, privat, hous, patio, pot, work, swim, seat, chair ,bbq, home, neighbour, privaci, entertain, read, exercis, kid, pet, pave, walk, eat, famili, furnitur,  coffe, peopl | | | |
|  | Estimate | Standard Error | P-value |  | Estimate | Standard Error | P-value |
| (Intercept) | -1.966 | 0.329 | 0.000 | (Intercept) | -0.870 | 0.340 | 0.011 |
| Connection to nature | 0.573 | 0.073 | 0.000 | Connection to nature | 0.126 | 0.075 | 0.093 |
| Age | 0.048 | 0.016 | 0.003 | Age | 0.007 | 0.017 | 0.663 |
| Gender (male) | -0.243 | 0.108 | 0.024 | Gender (male) | -0.060 | 0.114 | 0.600 |
| Income | -0.064 | 0.021 | 0.002 | Income | 0.001 | 0.022 | 0.969 |
| Education | 0.019 | 0.021 | 0.385 | Education | -0.048 | 0.022 | 0.031 |
| City (Sydney) | -0.223 | 0.106 | 0.035 | City (Sydney) | -0.093 | 0.112 | 0.406 |

**Supplementary note 2**

Participants were asked to select their age range (1 = 18-20 years (n = 62); 2 = 21-25 years (n = 170); 3 = 26-30 years (n = 142); 4 = 31-35 years (n = 200); 5 = 36-40 years (n = 191); 6 = 41-45 years (n = 209); 7 = 46-50 years (n = 162); 8 = 51-55 years (n = 172); 9 = 56-60 years (n = 166); 10 = 61-65 years (n = 176); 11 = 66-70 years (n = 194); 12 = more than 70 years (n = 240)). They were asked to select their gender (i.e., female (n = 1025); male (n = 1051); non-binary, other, prefer not to say (n = 8)). Participants were asked to select income range (i.e., 10 = $2000 or more a week (n = 436); 9 = $1,500 ‐ $1,999 a week (n = 270); 8 = $1,250 ‐ $1,499 a week (n = 184); 7 = $1,000 ‐ $1,249 a week (n = 222); 6 = $800 ‐ $999 a week (n = 153); 5 = $600 ‐ $799 a week (n = 148); 4 = $400 ‐ $599 a week (n = 173); 3 = $300 ‐ $399 a week (n = 84); 2 = $200 ‐ $299 a week (n = 44); 1 = $1 ‐ $199 a week (n = 49); 0 = Nil or negative income (n = 37); NA = Prefer not to say (n = 274)). Education level was recorded through following categories (1 = Year 8 or below (n = 8); 2 = Year 9 or equivalent (n = 23); 3 = Year 10 or equivalent (n = 109); 4 = Year 11 or equivalent (n = 28); 5 = Year 12 or equivalent (n = 248); 6 = Year 12 or equivalent (n = 220); 7 = Trade certificate (n = 98); 8 = Associate diploma (n = 121); 9 = Advanced diploma (n = 113); 10 = Bachelor degree (n = 680); 11 = Post‐graduate degree (n = 433), NA = other (n = 3)).

Items to measure connection to nature are

- I enjoy being outdoors, even in unpleasant weather.
- My ideal vacation spot would be a remote wilderness area.
- I enjoy digging in the earth and getting dirt on my hands
- I take notice of wildlife wherever I am.
- I don’t often go out in nature.
- The thought of being deep in the bush, away from civilization is frightening.

**Supplementary note 3**

1. About how often do you usually spend more than 10 minutes in your own yard or on your

deck? (please tick one)

 I don’t have a yard or deck

 Never

 Less than once a month

 2‐3 times a month

 Once a week

 2‐3 days a week

 4‐5 days a week

 6‐7 days a week

2. Thinking about the last week, about how much time in total did you spend in your own

yard or on your deck?

 No time

 1‐30 minutes

 31 minutes to 1 hour

 1‐3 hours

 3‐5 hours

 5‐7 hours

 7‐9 hours

 9+ hours

3. Describe your yard. What aspects do you like about it? What makes you want to spend time in it?

4. About how often do you usually visit or pass through outdoor greenspaces for any reason?

 Never

 Once a year

 Once every three months

 Once a month

 2‐3 times a month

 Once a week

 2‐3 days a week

 3‐5 days a week

 6‐7 days a week

5. Over the last week, what outdoor greenspaces did you visit or travel through? Can you

estimate the total time you spent there? (please list up to seven of the places you visited

for the longest time period). *This includes, for example, beaches, children's playgrounds, parks, bushland, bike‐ways, picnic areas, beaches, golf courses, tennis courts and bowling greens.*

| Name of Park (or location, e.g. cross streets) | How much time did you spend in it? |
| --- | --- |
|  |  |
|  |  |
|  |  |

6. Thinking about your favourite parks, or the ones you visit most, what are the aspects or factors that drive you to use these parks?

| Name of Park (or location, e.g. cross streets) | Factors that you like about the park, aspects that are attractive (open-ended) |
| --- | --- |
|  |  |
|  |  |
|  |  |
|  |  |
|  |  |

7. Please read each statement and check the box which indicates how much the statement applied to you *over the past week*. There are no right or wrong answers. Do not spend too much time on any statement.

|  | Did not  apply to  me at all | Applied to  me to  some  degree, or  some of  the time | Applied to me  to a  considerable  degree, or a  good part of  time | Applied to  me very  much, or  most of  the time |
| --- | --- | --- | --- | --- |
| I found it hard to wind down |  |  |  |  |
| I was aware of dryness of my mouth |  |  |  |  |
| I couldn't seem to experience any positive feeling at all |  |  |  |  |
| I experienced breathing difficulty (e.g. excessively rapid  breathing, breathlessness in the absence of physical  exertion) |  |  |  |  |
| I found it difficult to work up the initiative to do things |  |  |  |  |
| I tended to over‐react to situations |  |  |  |  |
| I experienced trembling (e.g. in the hands) |  |  |  |  |
| I felt that I was using a lot of nervous energy |  |  |  |  |
| I was worried about situations in which I might panic  and make a fool of myself |  |  |  |  |
| I felt that I had nothing to look forward to |  |  |  |  |
| I found myself getting agitated |  |  |  |  |
| I found it difficult to relax |  |  |  |  |
| I felt down‐hearted and blue |  |  |  |  |
| I was intolerant of anything that kept me from getting  on with what I was doing |  |  |  |  |
| I felt I was close to panic |  |  |  |  |
| I was unable to become enthusiastic about anything |  |  |  |  |
| I felt I wasn't worth much as a person |  |  |  |  |
| I felt that I was rather touchy |  |  |  |  |
| I was aware of the action of my heart in the absence of physical exertion (e.g. sense of heart rate increase,  heart missing a beat) |  |  |  |  |
| I felt scared without any good reason |  |  |  |  |
| I felt that life was meaningless |  |  |  |  |

8. Please rate the extent to which you agree with each statement. Please tick the box that

indicates how you really feel, rather than how you think “most people” feel.

| Statements | Disagree  strongly | Disagree  a little | Neither  Agree  nor  Disagree | Agree  a  little | Agree  strongly |
| --- | --- | --- | --- | --- | --- |
| I enjoy being outdoors, even in unpleasant weather. |  |  |  |  |  |
| My ideal vacation spot would be a remote wilderness area. |  |  |  |  |  |
| I enjoy digging in the earth and getting dirt on my hands. |  |  |  |  |  |
| I take notice of wildlife wherever I am. |  |  |  |  |  |
| I don’t often go out in nature. |  |  |  |  |  |
| The thought of being deep in the bush, away from civilisation, is frightening. |  |  |  |  |  |

9. What is your age range?

 18‐20 years

 21‐25 years

 26‐30 years

 31‐35 years

 36‐40 years

 41‐45 years

 46‐50 years

 51‐55 years

 56‐60 years

 61‐65 years

 66‐70 years

 70+ years

10. What gender do you identify with?

 Female

 Male

 Non-binary

 Other

 Prefer not to say

11. Before tax, what is the *total* of all wages/salaries, government benefits, pensions,

allowances and other income you *usually* receive? (please tick one)

 $2000 or more a week ($104,000 or more per year)

 $1,500 ‐ $1,999 a week ($78,000 ‐$103,999 per year)

 $1,250 ‐ $1,499 a week ($65,000 – $77,999 per year)

 $1,000 ‐ $1,249 a week ($52,000 ‐ $64,999 per year)

 $800 ‐ $999 a week ($41,600 ‐ $51,999 per year)

 $600 ‐ $799 a week ($32,200 ‐ $41,599 per year)

 $400 ‐ $599 a week ($20,800 ‐ $31,199 per year)

 $300 ‐ $399 a week ($15,600 ‐ $20,799 per year)

 $200 ‐ $299 a week ($1 ‐ $10,399 per year)

 $1 ‐ $199 a week ($1 ‐ $10,399 per year)

 Nil or negative income

 Prefer not to say

12. What is the level of the highest qualification or schooling year you have completed? (please tick one)

 Year 8 or below

 Year 9 or equivalent

 Year 10 or equivalent

 Year 11 or equivalent

 Year 12 or equivalent

 Certificate 1, 2, 3, 4

 Trade certificate

 Associate diploma

 Advanced diploma

 Bachelor degree

 Post‐graduate degree

 Other (please specify)________________________________________
